# Supplementary material for: Saccharomyces boulardii (CNCM I-745) improves intestinal damage in sepsis by remodeling intestinal flora structure
Source: Front Cell Infect Microbiol. 2025 Jul 31;15:1602792. doi: 10.3389/fcimb.2025.1602792 (PMC12350329; doi:10.3389/fcimb.2025.1602792)
Supplement: Supplementary file 1 [file DataSheet1.zip › summary/summary/7_Differential_analysis/1_diff_abundance/7_Species/Group.CW4_vs_AW4/Group.CW4_vs_AW4_diff_barplot.pdf]

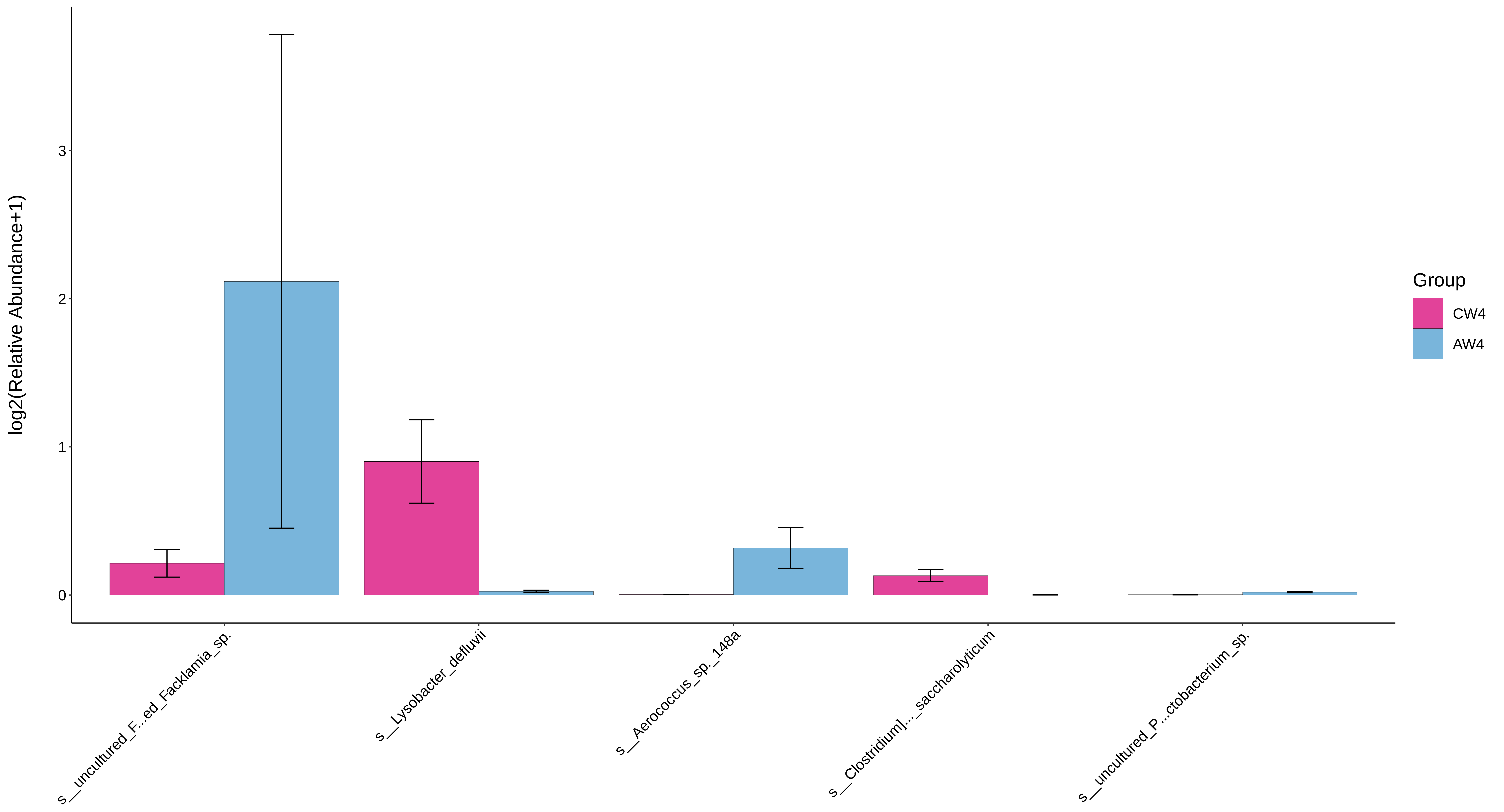

log2(Relative Abundance+1)
3
2
1
0
Group
CW4
AW4
s__uncultured_F...ed_Facklamia_sp.
s__Lysobacter_defluvii
s__Aerococcus_sp._148a
s__Clostridium]..._saccharolyticum
s__uncultured_P...ctobacterium_sp.
